# Tomato Pest Recognition Algorithm Based on Improved YOLOv4

**DOI:** 10.3389/fpls.2022.814681

**Published:** 2022-07-13

**Authors:** Jun Liu, Xuewei Wang, Wenqing Miao, Guoxu Liu

**Affiliations:** ^1^Shandong Provincial University Laboratory for Protected Horticulture, Blockchain Laboratory of Agricultural Vegetables, Weifang University of Science and Technology, Weifang, China; ^2^College of Information and Control Engineering, Weifang University, Weifang, China

**Keywords:** image processing, pests identification, YOLO, object detection, tomato

## Abstract

Tomato plants are infected by diseases and insect pests in the growth process, which will lead to a reduction in tomato production and economic benefits for growers. At present, tomato pests are detected mainly through manual collection and classification of field samples by professionals. This manual classification method is expensive and time-consuming. The existing automatic pest detection methods based on a computer require a simple background environment of the pests and cannot locate pests. To solve these problems, based on the idea of deep learning, a tomato pest identification algorithm based on an improved YOLOv4 fusing triplet attention mechanism (YOLOv4-TAM) was proposed, and the problem of imbalances in the number of positive and negative samples in the image was addressed by introducing a focal loss function. The K-means + + clustering algorithm is used to obtain a set of anchor boxes that correspond to the pest dataset. At the same time, a labeled dataset of tomato pests was established. The proposed algorithm was tested on the established dataset, and the average recognition accuracy reached 95.2%. The experimental results show that the proposed method can effectively improve the accuracy of tomato pests, which is superior to the previous methods. Algorithmic performance on practical images of healthy and unhealthy objects shows that the proposed method is feasible for the detection of tomato pests.

## Introduction

Agricultural pests are known to be one of the main factors causing damage to the world’s agricultural economy. As a kind of insect, they mainly depend on the survival of various plants and crops, causing different degrees of harm to agriculture, forestry, and animal husbandry. The economic impacts of agricultural pests spread worldwide. The economic losses of agriculture in Europe reached 28.2%, in North America reached 31.2%, and in Asia and Africa reached more than 50%. Since the 1960s, integrated pest control (IPM) (Parsa et al., 2014) has been the main pest control mode. IPM has formulated the best pesticide recommendations for economic development and ecological maintenance based on the results of pressure detection of different pests. Therefore, the accurate identification and location of pests are very important for IPM. At present, most detection methods are expensive and time-consuming because they require IPM professionals to collect and classify field samples manually, which prevents the developing countries that lack IPM technological support from using these technologies for pest control. Therefore, in the field of IPM, a fast and low-cost automatic detection method for agricultural pests is urgently needed.

In recent years, deep learning has developed rapidly and has attracted an increasing number of researchers’ attention because of its superior performance in feature extraction, model generalization, and fitting. The convolutional neural network (CNN) in the deep learning method performs well in large-scale image recognition tasks. The biggest difference between CNN and traditional pattern recognition methods is that it automatically extracts features layer by layer from images, which can contain thousands of parameters.

At present, many pest recognition systems have been proposed by researchers. Yang et al. (2017) proposed an insect recognition model based on deep learning and image saliency analysis. On the test set of tea garden images, the average accuracy was 0.915, the running time was reduced to 0.7 ms, and the required memory was 6 MB. Shen et al. (2018) used deep neural network technology to establish the detection and recognition method of stored grain pests. Faster R-CNN was used to extract the possible insect areas in the image and classify the insects in these areas. The average accuracy was 88%. Mique and Palaoag (2018) used a CNN-based model to retrieve and compare the collected images with a pile of rice pest images. The model can achieve 90.9% of the final training accuracy. Zhong et al. (2018) designed and implemented a vision-based classification system for flying insect counting. First, yellow sticky traps were set up in the monitoring area to trap flying insects, and a camera was set up to capture images in real-time. Then, a method of object detection and rough counting based on YOLO was designed, and a support vector machine based on global features was designed. Finally, six kinds of flying insects, including bees, flies, mosquitoes, moths, scarabs, and fruit flies, were selected to evaluate the effectiveness of the system. Compared with the conventional method, the experimental results show that the method performs better, and the average classification accuracy is 90.18%. Barbedo and Castro (2019) studied the effect of image quality on the identification of psylla using CNN. A total of 1,276 images were used in the experiment. Half of them were collected using a flat panel scanner, and the other half by two different brands of smartphones. The accuracy was 70 and 90%, respectively, which shows that a more realistic environment can guarantee the robustness of the trained network. He et al. (2020) built a brown rice planthopper detection model based on deep learning and achieved good results through the improvement of faster RCNN and YOLOv3 models. The authors compared these two models under equivalent conditions and showed that the YOLOv3 model performs better and has a higher detection rate than the faster RCNN. Liu et al. (2020) fused semantic information (temperature, humidity, longitude and latitude, etc.) of pest images with CNN models and verified the advantages of the attention mechanism in solving the problem of imbalanced data.

In this study, an algorithm that can diagnose tomato pests quickly and effectively by improving the YOLO model is proposed. It can solve the problem of low diagnostic accuracy of pests encountered by tomato producers during cultivation, and has some implications for future research on tomato pest prevention, and advance the development of intelligent agriculture.

## Related Works

### Object Detection

Object detection refers to recognizing the corresponding object category, location, and size from a given image or video, to carry out the next analysis. Object detection algorithms based on regression do not need to generate branches from candidate regions. For a given input image, the candidate boxes and categories of objects are directly regressed at multiple positions of the image. Therefore, this research will adopt the object detection algorithm based on regression.

In 2016, the YOLO network was proposed by Redmon et al. (2016). Based on YOLO, YOLOv2 (Redmon and Farhadi, 2017), YOLOv3 (Redmon and Farhadi, 2018), and YOLOv4 (Bochkovskiy et al., 2020) were proposed. The YOLO network, as a new and outstanding object detection technology, has been widely recommended by scholars. It needs only one neural network to detect objects. YOLO can read the whole image at a time and can recognize the local information of the image, which greatly reduces the false detection rate of the background. It has a slight decrease in accuracy compared with the most popular network, but it has a great improvement in speed. Fast YOLO has a speed of 155 frames per second, which can be well applied in the scenes with high real-time requirements. At present, YOLO has different versions, with YOLOv4 being much faster than the other versions in speed.

With the deepening of research on object detection, scholars apply the improved YOLO algorithm to the real-time detection of vehicles (Zhou et al., 2020), pedestrians (Xu et al., 2022), traffic signs (Zhou et al., 2020), ships (Tang et al., 2021), fruits (Wang and He, 2021), and so on. In addition, its application in the field of agricultural pest detection also began to appear. Zhong et al. (2018) designed a vision-based flying insect counting and classification system based on YOLO. The average counting accuracy of raspberry peel was 92.50%, and the average classification accuracy was 90.18%. He et al. (2020) proposed a rapid and accurate detection algorithm for brown rice planthopper, Yolov3. The average recall rate was 49.60%, and the average accuracy rate was 96.48%. Zha et al. (2021) proposed the YOLOv4_ MF model to detect forestry pests. The experimental results showed that compared with the YOLOv4 model, the mAP of the proposed model was 4.24% higher. Xin and Wang (2021) used YOLOv4 to test and verify images after quality level classification, and the recognition accuracy was 95%, which was much higher than the basic 84% of the DCNN model.

Compared with other CNN networks that use sliding classifiers, YOLO is a unified network that can simultaneously predict the location, size, and category of objects. It is a real-time object detection system based on a deep convolution neural network. As the YOLO network has the characteristics of end-to-end, the whole training and detection process from data input to result in output is completed in the network model, so it can guarantee accuracy and show a faster detection speed. So, this study combines the idea of YOLOv4 to detect pests.

### Attention Mechanism

Attention mechanisms play an important role in human perception (Corbetta and Shulman, 2002). An important property of the human visual system is that the entire scene cannot be processed simultaneously. Instead, to better capture the visual structures, humans utilize a range of local saccades and selectively focus on the salient parts (Zheng et al., 2015).

The introduction of attention mechanisms into CNN networks has recently been proposed in the field of object detection to improve performance on large-scale classification tasks. Wang et al. (2017) proposed a residual attention network using an encoder attention module. By refining the feature maps, the network can’t only perform well but also be robust to noise inputs. Hu et al. (2018) introduced a compact attention feature extraction library using global average pooling features to calculate the information weight of channel attention. Woo et al. (2018) used an efficient architecture that simultaneously utilizes spatial and channel attention modules to focus on more information, and excellent results have been achieved. Ju et al. (2021) introduced the attention mechanism into the YOLO algorithm, and the detection accuracy has been improved. Inspired by this, this study combines the YOLO algorithm with the attention module to do further research.

### The Aim of This Study

With the advancement of agricultural intelligence, object detection has achieved certain development in the agricultural field. At present, many deep learning methods for object detection are widely used in crop identification, long-range potential as well as pests and diseases detection, weed identification, fruit and vegetable quality detection, and automatic picking.

The pests that often occur in tomatoes include whiteflies, aphids, and leafminers. Once they occur, they will cause a lot of loss. Therefore, it is of great significance to identify tomato pests in order to control them in time and eliminate them in germination. The actual environment of tomato pest identification is very complex. To achieve a more effective and widely applicable pest detection technology and meet the needs of using the least and most convenient operation to complete expert-level pest detection, this study combines deep learning with tomato pest detection. To achieve the goal of rapid and highly accurate detection of images of tomato pests, this study proposed a deep learning model that is fast and can perform multi-object detection based on YOLOv4 and improved it by fusing the triplet attention (Song et al., 2018) mechanism. Experiments showed that the proposed model greatly improved the comprehensive detection ability of the images of tomato pests.

## Methodology

### Principle of YOLO

The YOLO algorithm treats the detection problem of an object as a regression problem of position coordinate and confidence score directly. Therefore, the YOLO algorithm can predict the category and location of multiple objects in real-time at one time. Unlike traditional object detection algorithms, which select the sliding window method and the Faster R-CNN algorithm to extract candidate regions, YOLO directly inputs the whole image into the network model for training and detection. This idea greatly improves the training and detection speed of the network model.

YOLOv4 is the fourth version of the YOLO series of algorithms. The first major improvement of the YOLOv4 model is to use CSPDarknet53 as its backbone network. CSPDarknet53 is mainly composed of the CBM module and CSP module. The CBM module is composed of the Conv, batch normalization (BN), and Mish activation functions. The CSP module contains two branches; one is the convolution of the main cadres. One is used to generate a large residual edge, which enhances the learning ability of CNN by splicing two branches across different levels and integrating channels. Another major improvement of YOLOv4 is that in the detection section, a spatial pyramid pooled layer SPP module is used, which enables any size of feature map to be converted to a fixed size feature vector, inherits the YOLOv3 approach in the prediction of the boundary box, generates *a priori* box of different scales using K-means clustering, and predicts on the feature map at different levels. The difference is that it uses the idea of PANet to fuse features at different levels.

In addition, YOLOv4 introduces mosaic augmentation. Its principle is to randomly select four images at a time and randomly scale, flip horizontally, flip vertically, and change the color gamut of the images. Then, according to a certain proportion, the four images are intercepted and stitched into a new training image. Because many objects in the real natural environment are not the detection target as the detection background, they will seriously affect the accuracy of the algorithm. So a mosaic is used to enrich the background of the detection object, which is conducive to the weight distribution of different characteristics of different pests in the training algorithm.

### Triplet Attention Module

**FIGURE 1:**
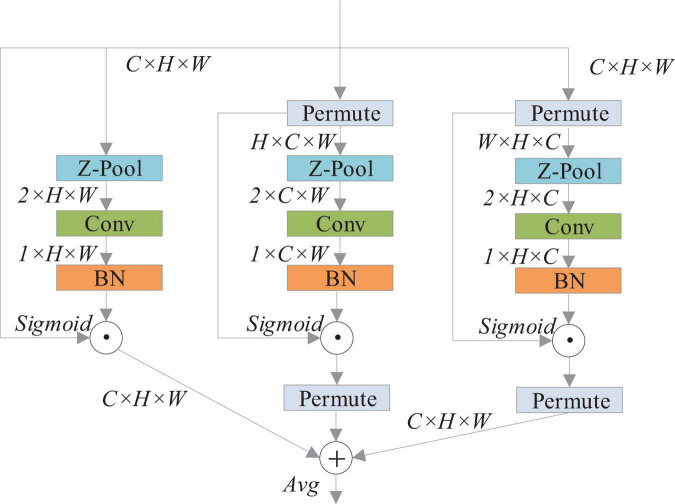
Network structure diagram of triplet attention (Song et al., 2018).

The YOLOv4 network treats the characteristics of each channel equally, which limits the detection performance of the algorithm to some extent. The tomato pest image background is complicated, and some pest targets are small in the area occupied by the image, which can easily cause misdetection. Therefore, the improvement of YOLOv4 is needed. To further improve the model accuracy, this study uses triplet attention to improve the CSPDarknet53 feature extraction network in YOLOv4. The triplet attention module (Song et al., 2018) is an inexpensive and effective attention mechanism with few parameters and does not involve dimensionality reduction. It is an additional neural network, as shown in Figure 1.

The triplet attention module consists of three parallel branches, two of which capture cross-dimensional interactions between channel C and space H or W. The last branch is used to build spatial attention. The output of the final three branches is aggregated on average.

**FIGURE 2:**
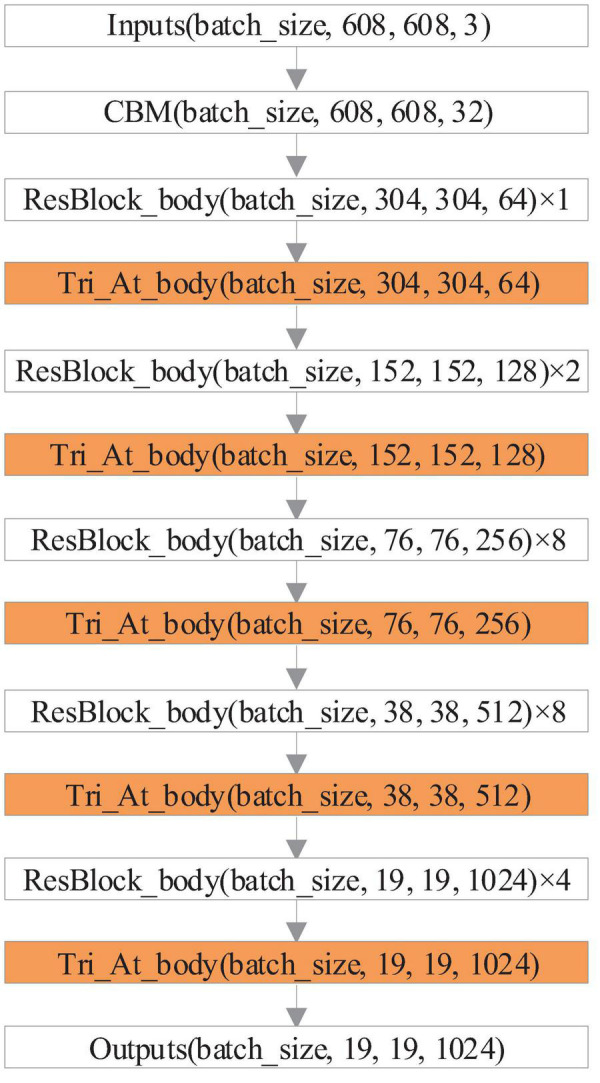
Network structure diagram of the proposed model.

This study uses the triplet attention module to improve the CSPDarknet53 network of YOLOv4, enabling the network to acquire cross-dimensional interactions through automatic learning, increasing effective feature channel weights, and thus making the network focus on important feature channels. The backbone network structure of the YOLOv4 model improved with the triplet attention module (YOLOv4-TAM) is shown in Figure 2.

### The New Loss Function

During the loss value calculation in YOLOv4, the detector divides the prediction box into positive and negative samples. The predicted box with the largest IOU value from the annotated box is divided into positive samples, and predicted boxes with all annotated boxes having IOU less than 0.5 are classified as negative samples. The small object occupies far fewer pixels in the image than the background does, resulting in a large difference in the number of positive and negative samples during training.

To this end, this study addresses the problem of imbalances in the number of positive and negative samples in the image by introducing a focal loss function, which is shown in the following formula:


$$L\,o\,s\,s=L\,o\,s\,s_{c\,o\,o\,r\,d}+L\,o\,s\,s_{o\,b\,j}+L\,o\,s\,s_{c\,l\,a\,s\,s}$$

$$=\lambda_{c\,o\,o\,r\,d}\,\sum_{i=0}^{S^{2}}\sum_{j=0}^{B}l_{i\,j}^{o\,b\,j}\,\left(2-w_{i}\times h_{i}\right)\,\left(1-C\,I\,O\,U\right)-$$

$$\lambda_{o\,b\,j}\sum_{i=0}^{S^{2}}\sum_{j=0}^{B}l_{i\,j}^{o\,b\,j}|C_{i}-{\hat{C}}_{i}{|}^{\beta }\cdot [\alpha {\hat{C}}_{i}\log\left(C_{i}\right)+\left(1-\alpha \right)\left(1-{\hat{C}}_{i}\right)$$

$$\cdot \log\left(1-C_{i}\right)]-\lambda {}_{n\,o\,o\,b\,j}\sum_{i=0}^{S^{2}}\sum_{j=0}^{B}l_{i\,j}^{n\,o\,o\,b\,j}|C{}_{i}-\hat{C}{}_{i}|{}^{\beta }\cdot [\alpha {\hat{C}}_{i}\log\left(C_{i}\right)$$

$$+(1-\alpha )\left(1-{\hat{C}}_{i}\right).\log(1-C_{i})]-\lambda_{obj}\sum_{i=0}^{S^{2}}\sum_{c\in class}l_{ij}^{obj}$$

(1) $$\left[{\hat{p}}_{i}\,\left(c\right)\,\log\left(p_{i}\,\left(c\right)\right)+\left(1-{\hat{p}}_{i}\,\left(c\right)\right)\,\log\left(1-p_{i}\,\left(c\right)\right)\right]$$

In the abovementioned formula, λ_*coord*_ is the weight coefficient of the coordinate prediction. *w_i_* and *h_i_* are the width and height of the annotation box, respectively. Complete intersection over union (CIOU) is a new IOU that has added the penalty coefficient of the annotation box and the predicted box. λ_*obj*_ is the weight coefficient when there is an object. λ_*noobj*_ is the weight coefficient when there is no object. α is used to balance positive and negative sample numbers, and this study takes the value of 0.75. β is used to moderate the weight of difficult and simple samples, and this study takes the value of 2. *S*^2^ is the number of grids. *B* is the number of predicted boxes in each grid.${\hat{C}}_{i}$ and *C_i_* are the confidence scores of the predicted box vs. true box, respectively.${\hat{p}}_{i}\,\left(c\right)$ and *p*_*i*_(*c*) are the probability values for the category of the predicted box vs. true box, respectively. $l_{i\,j}^{n\,o\,o\,b\,j}$ indicates that the object does not belong to the j bounding box of the i grid. $l_{i\,j}^{o\,b\,j}$ indicates that the object belongs to the j bounding box of the i grid.

### The New Anchor Boxes

Since the original YOLOv4 network was experimented on the VOC dataset, the original anchor box mechanism was set for the VOC dataset. For pest detection, utilizing the original anchor box mechanism would affect the IOU value, resulting in the inability to screen out the optimal prediction box. Therefore, the anchor box mechanism in the original YOLOv4 network needs to be improved. The K-means + + clustering algorithm can randomly generate clustering centers, which ensures a discrete type of initial cluster center, elevating the effect of anchor box generation. So the K-means + + clustering method is used to randomly choose the center of the sample and locate the anchor box for pest images. The new anchor boxes are obtained, including (13, 15), (19, 22), (23, 28), (44, 49), (52, 56), (64, 67), (87, 93), (102, 116), and (126, 139).

## Experiments

**FIGURE 3:**
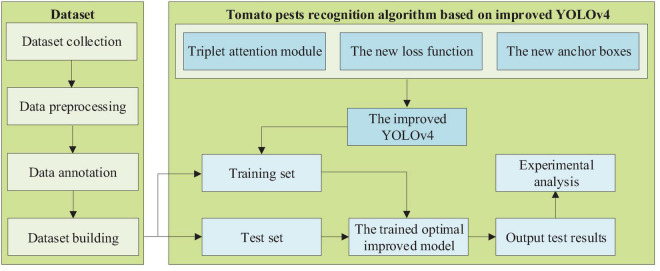
The experimental step flow of the study.

The experimental step flow of the study is shown in Figure 3.

### Dataset Collection

**FIGURE 4:**
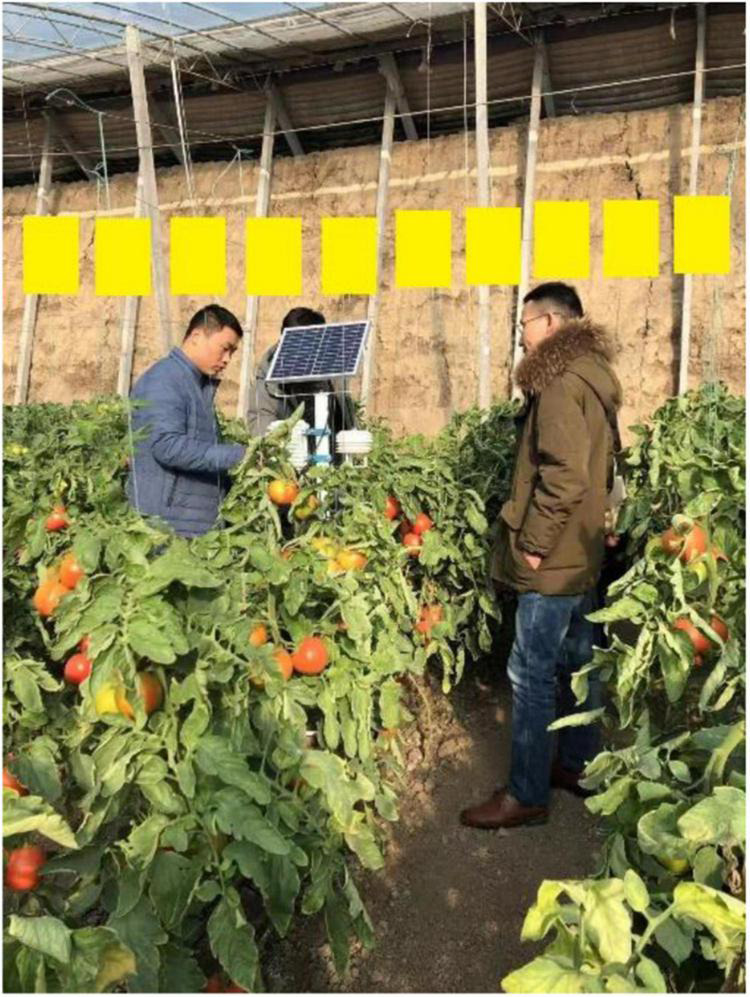
The experimental image acquisition site.

The main pests harming tomatoes in greenhouses are whiteflies, aphids, and leafminers. The pest image acquisition apparatus was installed in the Shouguang tomato greenhouse (36.8N, 118.7E) for this experiment (Figure 4). The yellow insect induction plate was utilized to attract the pests according to the principle of pest chemotaxis, and then the pests were glued by the high viscosity on the plate to achieve the trapping effect, by timed photographing the image of the insect induction plate and transmitting the image to the computer PC end for processing.

The image acquisition time of pests was from 22 October 2019 to 30 December 2020, and the species of pests captured by the induced insect plate were comprehensive and large in number. A total of 10 mutagen plates with a length of 35 cm and a width of 25 cm were suspended in the greenhouse and replaced every 5 days, and images of the mutagen plates were captured using an image acquisition device. The acquired image size was 1,960 × 1,080, and the image storage format was jpg. To make the experiment more closely resemble the real farm environment, all images were taken under natural conditions, and the adhered pests on the induced plate were cleaned up regularly by a dedicated person. A total of 2,893 images of induced plate pests were acquired for this experiment.

### Data Pre-processing

To further enrich the sample data while making up for the size and distribution limitations of pest targets and allow the model to achieve a better training effect, this study preprocessed the sample data. Mosaic, image rotation, multiscale cropping and magnification, image translation, image mirroring, and image denoising were used for data enhancement. After data pre-processing, the position distribution situation of the pest targets was enriched, and the small-size targets were enlarged to some extent, thus improving the generalization ability and training efficiency of the model.

### Data Annotation

**FIGURE 5:**
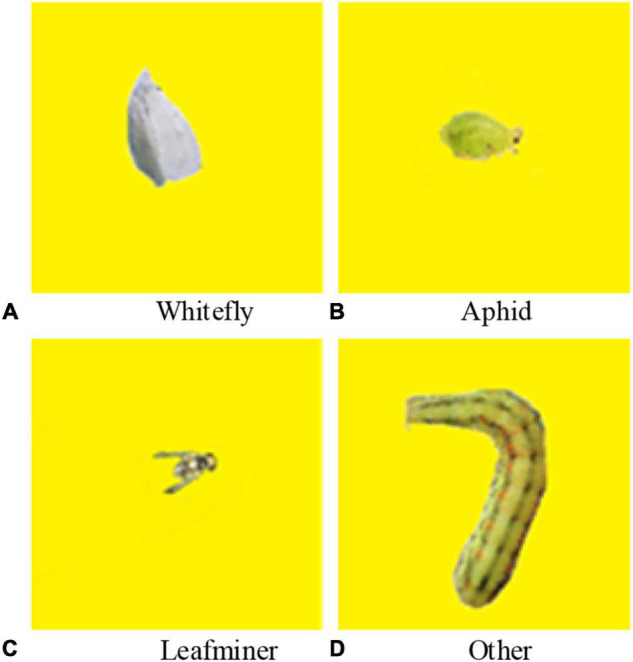
Examples of input images used in this study.

This experimental label was mainly divided into 4 categories, which were whiteflies, aphids, leafminers, and other large pests. The main purpose of classifying other large pests into one category was to explore the potential pest outbreak because large pests have a strong migration ability and are prone to large pest invasions in real-life production, which can increase the stress resistance of the algorithm when applied in practice. The sample number of pests in the image of the induced insect plate is huge, the situation when the occurrence of pests adhesion leads to an unclear separation is much lower than the situation when the pests are at an independent stage, and the removal of the number of the attached pests in the actual production does not affect the overall induced insect plate pests warning, so this study will only label the independent pests. The images were annotated using labeling, and the number of samples of whiteflies, aphids, potential leaf flies, and other large pests was 6,327, 5,687, 6,912, and 6679, respectively, as shown in Table 1 and Figure 5. Finally, 70% of images were randomly selected to construct the training set, 20% of images were used as the verification set, and the remaining images were used as the test set.

**TABLE 1:** Information on tomato pest dataset.

Class	Pests class	Labeling quantity
1	Whitefly	6327
2	Aphid	5687
3	Leafminer	6912
4	Other	6679

### Experimental Operation Environment

To better evaluate the performance of the proposed algorithm, it was compared with other pest recognition algorithms based on existing popular object detection methods, including DPM, R-CNN, Fast R-CNN, Faster R-CNN, and SSD, and the simulation platform configuration is shown in Table 2.

**TABLE 2:** Configuration of an experimental platform.

Server	CPU Processor: INTEL I7-9800X
	GPU: GEFORCE GTX1080Ti
	Memory: The Kingston 32G DDR4
Software	Operating System: Ubuntu 18.04
	Language: Python
	GCC 7.3.0
	CUDA 10.0.130
	OpenCV 3.4.5

*Among them, GPU acceleration was used for CUDA programming, and OpenCV was mainly used to display images during testing.*

### Evaluating Indicator

In the field of object detection, according to the research emphasis, the evaluation indexes can be different. The commonly used evaluation indexes include detection accuracy, efficiency, speed, positioning accuracy, and so on. This experiment mainly evaluates the model according to detection accuracy and detection speed.

(1) Detection accuracy

① mAP (mean average precision).

Usually, mAP is used as the evaluation criterion for detection accuracy. First, the average accuracy of each category in the dataset needs to be calculated as follows:


(2) $$P_{a\,v\,e\,r\,a\,g\,e}=\frac{1}{R}\,\sum_{j=1}^{n}I_{j}\cdot \frac{R_{j}}{j}$$

In the above formula, *R* represents the number of objects related to a category in the dataset (including detected and undetected), and *n* represents the number of objects in the dataset. If object *j* is relevant, then *I*_*j*_=1; if object *j* is irrelevant, then *I*_*j*_=0.*R*_*j*_ represents the number of related objects in the first *j* objects. Then the average of the average precision of multiple categories is taken as mAP:


(3) $$m\,A\,P=\frac{P_{a}\,v\,e\,r\,a\,g\,e}{N_{\left(c\,l\,a\,s\,s\right)}}$$

*N*(*class*) represents the number of all the categories. The larger the mAP value, the higher the monitoring accuracy of the algorithm; conversely, the lower the accuracy of the algorithm.

② Average precision (AP).

First, we need to introduce the precision-recall (PR) curve: the horizontal axis recall of the PR curve represents the ability of the classifier to cover the positive samples; the vertical axis precision represents the accuracy of the classifier to predict positive samples. Then the PR curve represents the trade-off between the accuracy of recognition of positive cases and the coverage ability of positive cases. AP is the area of the image enclosed by the PR curve and the horizontal axis.

For continuous PR curves:


(4) $$A\,P=\int_{0}^{1}P\,R\,dr$$

For discrete PR curves:


(5) $$A\,P=\sum_{k=1}^{n}P\,k\,\Delta \,r\,k$$

(2) Detection speed

Frames per second (FPS) is used to evaluate the detection speed. The more the FPS, the faster the detection speed of the algorithm is, otherwise, the slower the detection speed of the algorithm is.

## Experimental Results and Analysis

### Model Training

**FIGURE 6:**
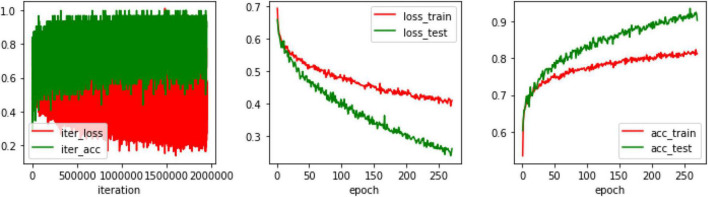
Process of model training.

Before training on the model, some initial settings are required. The values of hyperparameters must first be determined. In this experiment, the value of the batch is set to 32, and the value of the subdivisions is set to 16. That is, 2 images are passed into the network each time, 32 images are processed, and the model is updated and trained again with parameters. So, one epoch is for every 32 images. The learning rate is set to 0.0001, the weight delay is set to 0.0005, and the momentum is set to 0.9. After the first training, the prediction result of the network is not ideal enough. Through training with multiple epochs, a satisfactory training effect is produced. Figure 6 shows the training process. It can be seen that after training with 200 epochs, the loss of the network model decreases and stabilizes in a stepwise manner, i.e., a relatively satisfactory effect can be achieved after 200 epochs, and the training is continued in the experiment until the loss convergence is close to 0.

### Performance Comparison of Different Object Detection Algorithms

The experiment was carried out on the Darknet53 network. Faster R-CNN, SSD, YOLOv3, YOLOv4, and the proposed algorithm are the comparison algorithm. The five network model parameters are initialized by using the pre-training network model.

As shown by comparing the proposed algorithm with the other five algorithms in Table 3, the detection accuracy of the proposed algorithm is better than the other algorithms. Furthermore, in terms of detection speed, the proposed algorithm has an absolute advantage, which shows that the proposed algorithm can effectively carry out real-time detection.

**TABLE 3:** Comparison of training results of six models.

Object detection algorithms	mAP	FPS
Faster R-CNN	68.7	9
SSD	72.3	43
YOLOv3	73.6	71
YOLOv4	87.1	82
The proposed algorithm	93.4	83
		

Table 4 shows the proportion of detection errors for the six algorithms, with the proposed algorithm having the lowest error detection rate, only 0.36%. In consequence, the proposed algorithm in this study has a low false detection rate.

**TABLE 4:** Proportion of detection errors (%) for the six algorithms.

Algorithms	Number of false checks	Misdetection rate/%
Faster R-CNN	190	1.27%
SSD	65	0.43%
YOLOv3	71	0.47%
YOLOv4	63	0.42%
The proposed algorithm	54	0.36%
		

### Algorithmic Performance on Practical Images of Healthy and Unhealthy Objects

The algorithmic performance on practical images of healthy and unhealthy objects is shown in Table 5.

**TABLE 5:** Algorithmic performance on practical images of healthy and unhealthy objects.

Pests class	AP (%)
Whitefly	84.7
Aphid	83.9
Leafminer	62.7
Other	89.6
mAP (%)	78.1

As shown in Table 5, the AP of other pests is the highest and reaches 89.6%. However, the AP of leafminers is the lowest and only reaches 62.7%. The main reason for the large difference in detection accuracy between the two pests is the difference in pest image samples. The bodies of other pests are relatively large, and the number of pests in a single image is less, whereas the bodies of leafminers are relatively small, the number of pests in a single image is greater, and many are stacked together, resulting in greater detection difficulty and smaller AP. The mAP of the four pests reaches 78.1%, which has met the accuracy requirements of practical application and which shows that the proposed method is feasible for the detection of pests.

**FIGURE 7:**
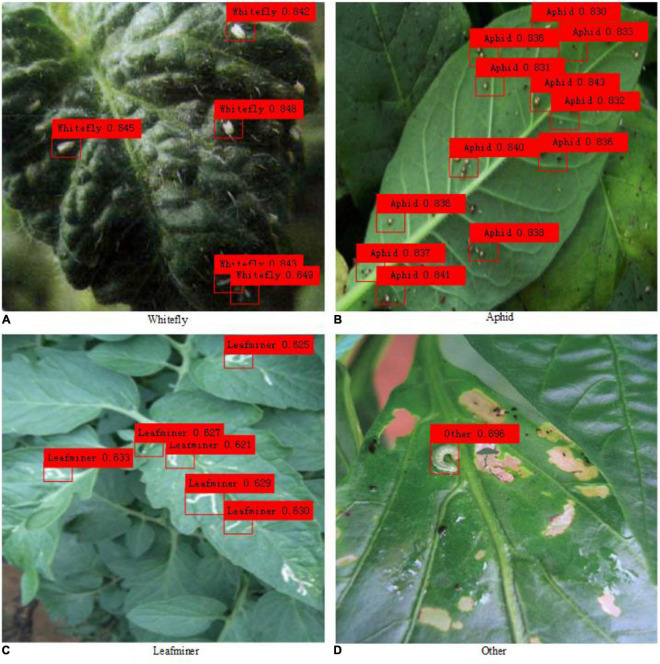
Detection effect of practical images of healthy and unhealthy objects.

The actual detection effect comparison of pest images is shown in Figure 7. The detection results of all pest objects in the figure are marked with color rectangular boxes. It can be seen intuitively that the proposed algorithm has better detection results for images with large pests, while for images with dense small pests, the pest detection results are slightly worse, and some pests cannot be detected.

## Conclusion and Future Directions

### Conclusion

In response to the problems of partial miss detection combined with poor detection accuracy that exists when using the YOLOv4 network to directly detect tomato pest images, this study proposes an improved YOLOv4 object detection method that employs a triplet attention mechanism and addresses the problem of imbalances in the number of positive and negative samples in the image by introducing a focal loss function. The experiment shows that the proposed model greatly improves the comprehensive performance on the image detection task of tomato pests based on not only increasing the complexity of the model on a small scale but also guaranteeing the real-time of the model, which is of great significance to reduce and prevent the incidence chance of tomato pests. Compared with other methods based on deep learning, this method can maintain high accuracy and has very prominent real-time performance, and can effectively identify the type and location of pests on the images with a small false detection rate and good robustness.

### Future Directions

Although good experimental results have been achieved in this study for image recognition research of tomato pests, it is of great significance for tomato pest prediction and control. Because of the limited time, other things need further research:

(1) Current research focuses on the processing of static images, and how image recognition techniques can be applied in videos, integrated with monitoring devices is something to be investigated next. The application of image recognition technology in videos requires that the algorithms process fast, have high accuracy rates, and have requirements such as automation, continuity, and so on. It is difficult to meet the requirements only with the computational quantity of current algorithms. Borrowing from pedestrian detection methods is a feasible direction and requires further research.
(2) The sample size of the tomato pest image dataset established in this study is relatively large or far from that of standard-scale image datasets frequently used by the deep learning community, and the dataset size should be greatly expanded in future studies. It is also evident that the manual method cannot be adopted for the annotation of datasets alone, but in combination with existing detection models to automatically annotate new pest images, followed by corresponding manual corrections so that the combination of machine and manual annotation can greatly reduce the cost and time of work. Then the optimization and boosting of the object detection model should be studied in terms of a sufficiently capacitated dataset.
(3) The study of new algorithms need further research. It can be found that scientific development must have been helical. New algorithms can drive innovation of the whole technology, but there is always a validity period. There are many other ways to effectively optimize the model that still need to be attempted. In addition, how to solve the problem of pest adhesion and reduce the detected repeat box in the follow-up work will be the next research direction.

## Data Availability Statement

The original contributions presented in this study are included in the article/supplementary material, further inquiries can be directed to the corresponding author.

## Author Contributions

JL and GL designed research and developed the detection dataset. JL and XW conducted the experiments, data analysis, and wrote the manuscript. GL and WM revised the manuscript. All authors read and approved the manuscript.

## Conflict of Interest

The authors declare that the research was conducted in the absence of any commercial or financial relationships that could be construed as a potential conflict of interest.

## Publisher’s Note

All claims expressed in this article are solely those of the authors and do not necessarily represent those of their affiliated organizations, or those of the publisher, the editors and the reviewers. Any product that may be evaluated in this article, or claim that may be made by its manufacturer, is not guaranteed or endorsed by the publisher.
